# Genetic Variants in Meiotic Program Initiation Pathway Genes Are Associated with Spermatogenic Impairment in a Han Chinese Population

**DOI:** 10.1371/journal.pone.0053443

**Published:** 2013-01-08

**Authors:** Chuncheng Lu, Miaofei Xu, Ying Wang, Yufeng Qin, Guizhen Du, Wei Wu, Xiumei Han, Chao Ji, Yanli Yang, Aihua Gu, Yankai Xia, Ling Song, Shoulin Wang, Xinru Wang

**Affiliations:** 1 State Key Laboratory of Reproductive Medicine, Institute of Toxicology, Nanjing Medical University, Nanjing, China; 2 Key Laboratory of Modern Toxicology of Ministry of Education, School of Public Health, Nanjing Medical University, Nanjing, China; 3 Department of Dermatology, the First Affiliated Hospital of Nanjing Medical University, Nanjing, China; 4 Department of Otorhinolaryngology, the First Affiliated Hospital of Nanjing Medical University, Nanjing, China; Sanjay Gandhi Medical Institute, India

## Abstract

**Background:**

The meiotic program initiation pathway genes (*CYP26B1*, *NANOS1* and *STRA8*) have been proposed to play key roles in spermatogenesis.

**Objective:**

To elucidate the exact role of the genetic variants of the meiosis initiation genes in spermatogenesis, we genotyped the potential functional genetic variants of *CYP26B1, NANOS1* and *STRA8* genes, and evaluated their effects on spermatogenesis in our study population.

**Design, Setting, and Participants:**

In this study, all subjects were volunteers from the affiliated hospitals of Nanjing Medical University between March 2004 and July 2009 (NJMU Infertile Study). Total 719 idiopathic infertile cases were recruited and divided into three groups according to WHO semen parameters: 201 azoospermia patients (no sperm in the ejaculate even after centrifugation), 155 oligozoospermia patients (sperm counts <20×10^6^/ml) and 363 infertility/normozoospermia subjects (sperm counts >20×10^6^/ml). The control group consisted of 383 subjects with normal semen parameters, all of which had fathered at least one child without assisted reproductive technologies.

**Measurements:**

Eight single nucleotide polymorphisms (SNPs) in *CYP26B1, NANOS1* and *STRA8* genes were determined by TaqMan allelic discrimination assay in 719 idiopathic infertile men and 383 healthy controls.

**Results and Limitations:**

The genetic variant rs10269148 of *STRA8* gene showed higher risk of spermatogenic impairment in the groups of abnormospermia (including azoospermia subgroup and oligozoospermia subgroup) and azoospermia than the controls with odds ratios and 95% confidence intervals of 2.52 (1.29–4.94) and 2.92 (1.41–6.06), respectively (*P* = 0.006, 0.002 respective). Notably, larger sample size studies and in vivo or in vitro functional studies are needed to substantiate the biological roles of these variants.

**Conclusions:**

Our results provided epidemiological evidence supporting the involvement of genetic polymorphisms of the meiotic program initiation genes in modifying the risk of azoospermia and oligozoospermia in a Han-Chinese population.

## Introduction

Infertility was reported affecting 10–15% of couples, and roughly half of which are due to the man’s problem [1], [2]. Spermatogenic impairment is the most common form of male infertility, which is closely related with impaired preimplantation development, low fertilization rate, increased abortion and elevated incidence of disease in the offspring, and as well as child cancer [3]. Spermatogenesis is a highly regulated process which can be subdivided into three main phases: mitotic proliferation, meiosis and sperm morphogenesis, among which, the meiosis is an important event in the process of sexual reproduction of biology gametes (including male gametes and female gametes) generation, and its smooth start is key to the final completion of meiosis. But we still lack a detailed understanding of the molecular mechanism for the initiation of germ cell meiosis [4], [5], [6], [7]. Recently, a series of studies have shown that several genes (*CYP26B1*, *NANOS1*, *STRA8 et al*) co-modify the process of meiotic program initiation [5], [6], [8], [9].

Of these genes, the *STRA8*, stimulated by retinoic acid gene 8, was activated by retinoic acid (RA), which can directly enter the nucleus and bind to the corresponding retinoic acid receptor (RAR) to regulate the expression of specific genes. RA plays an important role in the process of cell growth, differentiation and apoptosis, and is catabolized by a family of cytochrome P450 (CYP) enzymes, *CYP26A1*, *CYP26B1* and *CYP26C1* [10], [11], [12]. *CYP26B1* is expressed in not the embryonic ovary but the embryonic testis, implying that the *CYP26B1* is closely associated with male germ cell development. The expression of *CYP26B1* in the testis of wild-type mouse is increased at embryonic day (E) 11.5, prior to mitotic arrest, and then reduced at E13.5 [13]. The degradation effect of *CYP26B1* on *RA* concentration may inactivate *STRA8*, thus ensuring the normal development of spermatogonia. Previous study reports that genetic deletion of *CYP26B1* leads to increased *RA* levels and activation of *STRA8* in the embryonic testes. As a consequence, male germ cells are prematurely entering into meiosis, arrested at pachytene stage, causing a rapid increase in apoptosis and a lack of spermatogonia of male mice after birth [13], [14], [15], [16]. If no other *STRA8* inhibitory factors exist, the concentration of RA will be increased, and the *STRA8* will be re-activated. To ensure the process of meiosis initiation go normally, other inhibitory factors are required.

The *NANOS* (human *NANOS1*, mice *Nanos2* and *Nanos3*) is one of the evolutionarily conserved proteins implicated in germ cell development and closely related with somite and germ cell development [17]. It was reported the phenotype of *Nanos2* knockout mice’s testis during the embryonic period was similar to that of *Cyp26b1* functional defects [18]. It was interesting that *Stra8* expression in these two gene knockout mice showed certain period difference. In the testis of *Cyp26b1* knockout mice, it begins to express highly in embryonic E13.5, but appears at E14.5 and continues until after birth in Nanos2 knockout mice’s testis. It seems that the *Nanos2* begins to play an inhibitory effect on *Stra8* when the *Cyp26b1* expression decreases [6], [18]. The synergy of these three regulating factors avoids the early meiosis initiation of spermatogonial cells, which ensures the normal development of sperm [19]. Thus, *STRA8*, *CYP26B1, and NANOS1* were chosen as the typical meiotic program initiation pathway genes in this study.

Considering the essential role of meiotic program initiation pathway genes in spermatogenesis, we speculate that genetic variants of these genes have the potential to affect normal spermatogenesis. To test our hypothesis, we performed genotyping analyses for eight SNPs (rs2241057, rs707718, rs1422627, rs9304651, rs2015728, rs10269148, rs17168319 and rs17168337) in these genes, and investigated the association between these genetic variants and idiopathic infertility in a Chinese population. To the best of our knowledge, this is the first time to investigate the associations between these meiotic program initiation pathway gene polymorphisms and susceptibility to spermatogenic impairment.

## Materials and Methods

### Subject Recruitment and Sample Collection

The study was approved by the Ethics review board of Nanjing medical university. The protocol and consent form were approved by the Institutional Review Board of Nanjing Medical University prior to the study. All participants provided their written informed consent to join in this study. And all the subjects were genetically unrelated ethnic Han-Chinese from East China. Every participant received complete medical history questionnaire, physical examinations and semen analysis. All infertility patients were examined, among which, those with a history of Y chromosome microdeletions, cytogenetic abnormalities, congenital bilateral absence of vas deferens, cryptorchidism and orchitis were excluded [20]. Additionally, subjects having special occupational exposure which may be suspected to affect semen quality were precluded. After completing the questionnaire, each subject donated 5 ml of blood used for genomic DNA extraction.

### SNP Selection and Genotype Analyses

Through information gained from PubMed and Hapmap searches, we identified the potential functional polymorphisms in *CYP26B1, NANOS1* and *STRA8.* All selected single nucleotide polymorphisms (SNPs) have reported minor allele frequencies (MAF) of >0.05 in Pubmed and are located in exons and UTRs. In the case of multiple SNPs in the same haplotype block (linkage coefficient r^2^>0.8), only one was selected. Finally, we identified eight potential functional polymorphisms (rs2241057, rs707718, rs1422627, rs9304651, rs2015728, rs10269148, rs17168319 and rs17168337) in three genes involved in the meiotic program initiation (Table 1).

**Table 1:** The meiotic program initiation pathway genes and polymorphisms evaluated in this study.

Gene	SNP ID	Position	Nucleotide change	Amino acid change	MAF (℅)^a^	p value for HWE Test
Cyp26b1	rs2241057	nsSNP	T>C	Leu>Ser	11.0	0.552
	rs707718	3′UTR	A>C	–	45.3	0.878
Nanos2	rs1422627	3′UTR	T>C	–	30.2	<0.01
	rs9304651	5′near gene	A>G	–	9.8	0.472
	rs2015728	5′near gene	G>T	–	27.4	0.449
Stra8	rs10269148	5′near gene	C>G	–	3.5	0.735
	rs17168319	5′near gene	A>G	–	23.2	0.931
	rs17168337	3′near gene	C>G	–	31.1	0.386

Abbreviations: nsSNP, non-synonymous; UTR, untranslated region; MAF, minor allele frequency; HWE, Hardy-Weinberg equilibrium.

a Minimum allele frequency in the general Han Chinese population, as reported in dbSNP database.

Genomic DNA of the subjects was isolated from peripheral blood lymphocytes. These SNPs were genotyped by using TaqMan SNP allelic discrimination. For quality control, 10% of the samples were randomly genotyped again, and the reproducibility was 100%.

### Statictical Analysis

Statistical analyses were carried out using the Stata 10.0 statistical package (StataCorp LP, USA). The Hardy-Weinberg equilibrium (HWE) tests were performed on online software, SHEsis (http://analysis.bio-x.cn/myAnalysis.php). The subjects with idiopathic infertility were first divided into two groups according to semen parameters: the abnormospermia group with sperm count below the WHO reference value and the normozoospermia group with the normal sperm count at or above the WHO reference value. And then the abnormospermia group was further divided into two subgroups: the azoospermia group and the oligozoospermia group. The semen analysis was described previously [20]. Values for semen parameters were the mean of at least two analyses.

Analysis of variance (ANOVA) was used to compare the mean age, tea consumption, Body Mass Index (BMI) between the case and control groups. The chi-squared test was used to evaluate the differences in smoking status and drinking status of each SNP in the three genes between the case and control groups. Estimated infertility risks with odds ratios (OR) and 95% confidence intervals (95%CI) were calculated by multivariate logistic regression with adjustment for age, smoking status and BMI, where it was appropriate. Two-sided tests were used and the Bonferroni adjustment for multiple testing was applied. The applied *p*-value for a truly significant result is calculated as 0.05/n.

## Results

### Characteristics of the Study Populations

The final population consisted of 1102 Han Chinese subjects, composed of 383 fertile controls, 201 azoospermia, 155 oligozoospermia and 363 infertility/normozoospermia. The distributions of selected characteristics among the case and control subjects were presented in Table 2. All groups had similar patterns of drinking and tea consumption (*p*>0.05). The mean ages were higher in the groups of all infertility, azoospermia and oligozoospermia than the control group (all *p*<0.05). BMI levels in the group of all infertility were significantly higher than those in the control subjects. Smoking prevalence was higher in the azoospermia group than the control group (*p*<0.05).

**Table 2:** The distributions of selected variables among cases and control subjects.

Variables		Fertility/normozoospermia(n = 383)	Infertility/abnormospermia (n = 356)	Azoospermia (n = 201)	Oligozoospermia (n = 155)	Infertility/normozoospermia(n = 363)	All infertility(n = 719)
aAge, mean(SD)		29.79±3.55	29.22±4.50	29.55±4.78	28.79±4.08*	29.17±4.20*	29.19±4.35*
bSmoking	Ever	218(56.92)	184(51.69)	97(48.26) *	87(56.13)	193(53.17)	377(52.43)
	Never	165(43.08)	172(48.31)	104(51.74)	68(43.87)	170(46.83)	342(47.57)
bDrinking	Ever	214(55.87)	198(55.62)	114(56.72)	84(54.19)	179(49.31)	377(52.43)
	Never	169(44.13)	158(44.38)	87(43.28)	71(45.81)	184(50.69)	342(47.57)
Tea consumption	Ever	212(55.35)	176(49.44)	103(51.24)	73(47.10)	200(55.10)	376(52.29)
	Never	171(44.65)	180(50.56)	98(48.76)	82(52.90)	163(44.90)	343(47.71)
aBMI, mean(SD)		23.62±2.92	23.21±3.02	23.15±2.91	23.28±3.17	23.20±3.02	23.21±3.02*

a Independent-samples T-test for comparing the mean of the age, BMI and Pack-years of smoking between the cases and controls.

b Two-sided chi-squared test for either selected variable distributions between cases and controls.

* p<0.05 for two-sided chi-squared test for either selected characteristics distributions or allele frequencies between control and case group.

### Associations between Polymorphisms and Spermatogenic Impairment

The position and minor allele frequency of the 8 potential functional SNPs, were presented in Table 1. All SNPs frequencies were in accordance with HWE, except rs1422627 which had a *p* value <0.01 for deviation from HWE. The associations between meiosis initiation genes SNPs and the risks of male infertility were shown in Table 3. According to the Table 2, 95% CI was adjusted for the age, BMI and smoking in cases and controls because of its significant distribution differences.

**Table 3:** Associations of selected meiotic program initiation pathway gene polymorphisms and the risk of idiopathic male infertility.

	Genotype	Control^af^	Infertility^b^/abnormospermia			Azoospermia^c^			Oligozoospermia^d^			Infertility^e^/normozoospermia			All infertility		
		N	N	OR^f^ (95% CI)	p^g^	N	OR^f^ (95% CI)	p^g^	N	OR^f^ (95% CI)	p^g^	N	OR^f^ (95% CI)	p^g^	N	OR^f^ (95% CI)	p^g^
CYP26B1																	
rs2241057	TT	318	308	1.00	0.401	169	1.00	0.949	139	1.00	0.129	321	1.00	0.099	629	1.00	0.116
	CT	63	46	0.75(0.50–1.14)		31	0.96(0.60–1.53)		15	0.53(0.29–0.96)		40	0.61(0.40–0.94)		86	0.68(0.48–0.97)	
	CT/CC	65	48	0.76(0.51–1.14)	0.188	32	0.96(0.60–1.53)	0.746	16	0.54(0.30–0.98)	0.051	42	0.62(0.41–0.94)	0.035	90	0.68(0.48–0.97)	0.043
rs707718	AA	97	111	1.00	0.206	55	1.00	0.740	56	1.00	0.039	99	1.00	0.824	210	1.00	0.392
	AC	190	161	0.74(0.52–1.04)		93	0.88(0.58–1.33)		68	0.61(0.40–0.95)		177	0.91(0.64–1.29)		338	0.81(0.60–1.09)	
	AC/CC	286	245	0.75(0.54–1.03)	0.077	146	0.92(0.62–1.36)	0.594	99	0.59(0.40–0.89)	0.012	264	0.90(0.65–1.25)	0.546	509	0.81(0.61–1.08)	0.171
NANOS1																	
rs9304651	AA	330	297	1.00	0.579	164	1.00	0.347	133	1.00	0.975	306	1.00	0.741	603	1.00	0.600
	AG	50	56	1.24(0.82–1.88)		35	1.38(0.86–2.21)		21	1.08(0.62–1.87)		53	1.20(0.79–1.83)		109	1.21(0.84–1.74)	
	AG/GG	53	59	1.24(0.83–1.85)	0.300	37	1.38(0.87–2.19)	0.146	22	1.07(0.62–1.83)	0.914	57	1.22(0.81–1.84)	0.473	116	1.21(0.85–1.73)	0.314
rs2015728	GG	207	191	1.00	0.282	112	1.00	0.530	79	1.00	0.215	206	1.00	0.117	397	1.00	0.127
	GT	153	133	0.94(0.69–1.28)		73	0.88(0.61–1.26)		60	1.04(0.70–1.55)		124	0.83(0.61–1.12)		257	0.91(0.70–1.18)	
	GT/TT	176	165	1.02(0.76–1.36)	0.914	89	0.93(0.66–1.31)	0.699	76	1.15(0.79–1.67)	0.517	157	0.91(0.68–1.21)	0.458	322	0.98(0.76–1.26)	0.711
STRA8																	
rs10269148	CC	370	332	1.00	0.006	184	1.00	0.002	148	1.00	0.124	344	1.00	0.066	667	1.00	0.010
	CG	13	29	2.52(1.29–4.94)		19	2.92(1.41–6.06)		10	1.89(0.81–4.44)		23	1.86(0.93–3.74)		52	2.22(1.19–4.14)	
	CG/GG	13	29	2.52(1.29–4.94)	0.006	19	2.92(1.41–6.06)	0.002	10	1.89(0.81–4.44)	0.124	23	1.86(0.93–3.74)	0.066	52	2.22(1.19–4.14)	0.010
rs17168319	AA	217	188	1.00	0.500	104	1.00	0.440	84	1.00	0.847	210	1.00	0.701	398	1.00	0.908
	AG	143	148	1.19(0.88–1.62)		86	1.28(0.90–1.83)		62	1.13(0.76–1.68)		127	0.92(0.68–1.25)		275	1.04(0.80–1.35)	
	AG/GG	166	168	1.17(0.87–1.56)	0.293	97	1.25(0.89–1.76)	0.257	71	1.10(0.76–1.61)	0.602	153	0.95(0.71–1.28)	0.742	321	1.04(0.81–1.34)	0.678
rs17168337	CC	160	165	1.00	0.440	91	1.00	0.620	74	1.00	0.247	164	1.00	0.643	329	1.00	0.439
	CG	181	153	0.82(0.60–1.11)		92	0.91(0.63–1.31)		61	0.72(0.48–1.08)		161	0.86(0.63–1.17)		314	0.83(0.64–1.08)	
	CG/GG	223	191	0.83(0.62–1.11)	0.211	110	0.89(0.63–1.26)	0.417	81	0.78(0.53–1.14)	0.206	199	0.86(0.65–1.16)	0.349	390	0.84(0.65–1.08)	0.205

SNPs, single-nucleotide polymorphisms; OR, odds ratios; CI, confidence interval.

a Subjects consisted of proven fertility men with semen volume ≥2 ml, sperm counts ≥20×10^6^/ml and sperm motility ≥50% motile sperm.

b Subjects consisted of idiopathic infertile men with sperm counts <20×10^6^/ml.

c Subjects consisted of idiopathic infertile men with sperm counts = 0/ml.

d Subjects consisted of idiopathic infertile men with sperm counts from 0.1 to 20×10^6^/ml.

e Subjects consisted of idiopathic infertile men with sperm counts ≥20×10^6^/ml.

f ORs were obtained from multivariate logistic regression analysis.

g Two-sided χ2 test for genotype distributions between cases and controls.

Initially, we overviewed the frequency distribution between the total case group and the control group, and no significant difference was found. To investigate the possible role of the meiotic program initiation pathway genes in spermatogenesis, we subdivided the case group into two subgroups on the basis of sperm concentration: infertility/abnormospermia and infertility/normozoospermia. The genotype frequencies of rs10269148 in *STRA8* were significantly different existed between the infertility/abnormospermia group and the control group. Then the subgroup of infertility/abnormospermia was further stratified into two subgroups: the azoospermia group and the oligozoospermia group. Compared to the control group, statistically significant increased risk of idiopathic spermatogenic impairment was found for carriers of rs10269148CG genotype of *STRA8*, when compared with homozygous carriers of the C allele in the infertility/abnormospermia group and azoospermia subgroup respectively [ORs and 95% CIs being 2.52 (1.29–4.94), 2.92 (1.41–6.06)], while in the oligozoospermia group, the frequency distribution showed no significant difference. These results suggested that the G allele of rs10269148(C>G) may contribute to the severe idiopathic spermatogenic impairment.

To the rs2241057 of *CYP26B1*, although the distribution frequencies were lower in the total infertility group [12.52% (CT+CC)] than that in the control group [16.98% (CT+CC)], the distribution difference was not statistically significant after Bonferroni correction (p = 0.04>0.05/7 OR = 0.68, 95% CI = 0.48–0.97). And to the rs707718, logistic regression analysis revealed that compared with the rs707718 AA genotype, subjects in oligozoospermia group carrying the heterozygous rs707718 AC genotype had a significantly 39% decreased risk of spermatogenic impairment (OR = 0.61, 95% CI = 0.40–0.95). When we combined the rs707718 AC and CC genotype, assuming a co-dominant allele effect, the combined rs707718 AC+CC variant genotypes were associated with a 41% (OR = 0.59, 95% CI = 0.40–0.89) reduced risk of spermatogenic impairment. The OR values suggested that the variants rs2241057T>C, rs707718A>C may be protective factors against the risk of idiopathic male infertility with abnormal and/or normal semen parameters.

As to the other SNPs, no significant differences of distribution frequencies were identified among the case and control groups. In our study, due to the low occurrence frequency in Asians, we didn’t analyse homozygous mutant separately.

## Discussion

Although meiosis initiation genes have been recognized as key regulators in sperm function and male fertility [14], [18], [21], there have been few studies concerning the potential role of genetic variants of meiosis initiation genes in infertility, particularly idiopathic male infertility. *STRA8* is required for premeiotic DNA replication, while *CYP26B1* is decreased. By preventing *STRA8’*s expression, *CYP26B1* and *NANOS1* play critical roles in the differentiation of male germ cells [9], [13], [14], [22]. In all, the entry of testicular germ cells into meiotic program may be partly controlled by the stage-specific expression of *CYP26B1*and *NANOS1* expression in the germ cells [9], [13]. To investigate the exact role of meiotic initiation pathway genes, key downstream antagonists (*CYP26B1*, *NANOS1*) and effectors (*STRA8*) of the meiosis-inducing action of *RA* were analyzed in this study. Through information gained from PubMed and Hapmap searches, eight potential functional polymorphisms in meiosis initiation genes were examined.

*Vivo* studies have demonstrated that male mice lacking *STRA8* function produce no sperm [8], [23] mainly by hampering the homologous-chromosome pairing way [24]. But the exact functional genetic variants of this gene remain unclear. In this study, we found the rs10269148 significantly increased the risk of spermatogenic impairment associated with abnormal semen parameters (*p* = 0.006). In the following stratified analysis, compared with homozygous type CC, the rs10269148 C>G increased the risk of azoospermia and displayed 2.92 fold increased risk of spermatogenic impairment, which was consistent with the phenotypes in STRA8^−/−^ mice [8]. While in male mutant mice, the premeiotic DNA replication was conserved [8] and the germ cells could partly condense chromosomes, and initiate meiotic recombination until the leptotene stage of prophase I [24]. SNP rs10269148 is located in 5′-untranslated region of the STRA8 gene, and may affect gene function by altering transcription factor-binding sites, or the location, level and timing of gene expression [8], [24]. To clarify the precise mechanism, functional research should be conducted in the future. In fetal testis, the expression of *STRA8* is suppressed by a retinoid-degrading enzyme, which is mainly coded by *CYP26B1*. The *CYP26B1*, an important member of *CYP* family, is expressed in sertoli cells, and has a crucial role in the formation of sperm [9], [15], [16]. We found that individuals carrying rs2241057 genotype in the coding region of *CYP26B1*, which causes Leu to Ser substitution at position 264 (rs2241057 T>C) (http://www.ncbi.nlm.nih.gov/projects/SNP/snp_ref.cgi?rs=2241057), may lower the risk of spermatogenic impairment in our study population. As to the rs707718, comparing with homozygous type AA, the A>C mutation significantly reduced the risk of spermatogenic impairment, displaying 0.59 fold decreased risk of spermatogenic impairment (OR = 0.59, 95% CI = 0.40, 0.89, *p* = 0.012). However this difference disappeared after the Bonferroni correction. The statistical power of our study may be limited by the small sample size.

Our results were consistent with that the substitution of *CYP26B1* (rs2241057 T>C) enhanced the catabolicing activity of *CYP26B1* substantially, both in a cell free system and in cell culture. The actual catabolic rates in *vivo* depend on the local tissue concentration of atRA and many other factors, which cannot be accurately predicted from the data. But it is plausible that the differences between variants of *CYP* enzyme alter the equilibrium of etinoid synthesis and catabolism [25], [26]. Considering the function of *CYP26B1* in biogenesis and postnatal growth, we speculate that the genetic variation may affect the capacity of *CYP26B1* enzyme, then the meiotic program of germ cell, which might tightly associates with male infertility.

Although *CYP26B1* has an important inhibitory effect during the early initiation of meiosis in testicular germ cells, when it comes to E13.5, the expression begins to decrease [13], [15], which results in subsequent increase of RA concentration, thus producing the possibility of re-activation of *STRA8*. So the other *STRA8* inhibitory factors- *NANOS1* are required before the spermatogonial cells initiating meiosis. Given that *NANOS1* is an RNA-binding protein localized in the cytoplasm, we suspected the regulation of *STRA8* transcription may be an indirect effect of *NANOS1* [19]. In our study, we did not find any functional genetic variant of this gene. Thus, we speculated that the effect of the *NANOS1* gene on the *STRA8* gene might be moderate and other macromolecules might participate in the initiation of meiosis. The precise mechanism of the variants of *NANOS1* in male infertility needs further investigation.

In conclusion, the results of our study demonstrated for the first time that some representative genetic variants in the meiosis initiation genes might regulate the risk of male infertility. Although the statistical power of our study was limited by the small sample size in the subgroups, our findings might be helpful to understand the mechanism of male infertility. Larger sample size studies and in vivo or in vitro functional studies will be needed to substantiate the biological roles of these variants.

## References

[1] Okabe M, Ikawa M, Ashkenas J (1998) Male infertility and the genetics of spermatogenesis. American journal of human genetics 62: 1274–1281. 9644029 10.1086/301895 PMC1377172

[2] Dohle GR, Colpi GM, Hargreave TB, Papp GK, Jungwirth A, et al (2005) EAU guidelines on male infertility. European urology 48: 703–711. 16005562 10.1016/j.eururo.2005.06.002

[3] Brinkworth MH, Nieschlag E (2000) Association of cyclophosphamide-induced male-mediated, foetal abnormalities with reduced paternal germ-cell apoptosis. Mutation research 447: 149–154. 10751598 10.1016/s0027-5107(99)00189-x

[4] Pawlowski WP, Sheehan MJ, Ronceret A (2007) In the beginning: the initiation of meiosis. BioEssays : news and reviews in molecular, cellular and developmental biology 29: 511–514. 10.1002/bies.20578 17508404

[5] Bolcun-Filas E, Schimenti JC (2012) Genetics of meiosis and recombination in mice. International review of cell and molecular biology 298: 179–227. 22878107 10.1016/B978-0-12-394309-5.00005-5

[6] Ohta K, Lin Y, Hogg N, Yamamoto M, Yamazaki Y (2010) Direct effects of retinoic acid on entry of fetal male germ cells into meiosis in mice. Biology of reproduction 83: 1056–1063. 20826729 10.1095/biolreprod.110.085787 PMC2994329

[7] Kocer A, Reichmann J, Best D, Adams IR (2009) Germ cell sex determination in mammals. Molecular human reproduction 15: 205–213. 19218284 10.1093/molehr/gap008 PMC2657314

[8] Anderson EL, Baltus AE, Roepers-Gajadien HL, Hassold TJ, de Rooij DG, et al (2008) Stra8 and its inducer, retinoic acid, regulate meiotic initiation in both spermatogenesis and oogenesis in mice. Proceedings of the National Academy of Sciences of the United States of America 105: 14976–14980. 18799751 10.1073/pnas.0807297105 PMC2542382

[9] Koubova J, Menke DB, Zhou Q, Capel B, Griswold MD, et al (2006) Retinoic acid regulates sex-specific timing of meiotic initiation in mice. Proceedings of the National Academy of Sciences of the United States of America 103: 2474–2479. 16461896 10.1073/pnas.0510813103 PMC1413806

[10] Tahayato A, Dolle P, Petkovich M (2003) Cyp26C1 encodes a novel retinoic acid-metabolizing enzyme expressed in the hindbrain, inner ear, first branchial arch and tooth buds during murine development. Gene expression patterns : GEP 3: 449–454. 12915310 10.1016/s1567-133x(03)00066-8

[11] White JA, Guo YD, Baetz K, Beckett-Jones B, Bonasoro J, et al (1996) Identification of the retinoic acid-inducible all-trans-retinoic acid 4-hydroxylase. The Journal of biological chemistry 271: 29922–29927. 8939936 10.1074/jbc.271.47.29922

[12] White JA, Ramshaw H, Taimi M, Stangle W, Zhang A, et al (2000) Identification of the human cytochrome P450, P450RAI-2, which is predominantly expressed in the adult cerebellum and is responsible for all-trans-retinoic acid metabolism. Proceedings of the National Academy of Sciences of the United States of America 97: 6403–6408. 10823918 10.1073/pnas.120161397 PMC18615

[13] Bowles J, Knight D, Smith C, Wilhelm D, Richman J, et al (2006) Retinoid signaling determines germ cell fate in mice. Science 312: 596–600. 16574820 10.1126/science.1125691

[14] MacLean G, Li H, Metzger D, Chambon P, Petkovich M (2007) Apoptotic extinction of germ cells in testes of Cyp26b1 knockout mice. Endocrinology 148: 4560–4567. 17584971 10.1210/en.2007-0492

[15] Ross AC, Zolfaghari R (2011) Cytochrome P450s in the regulation of cellular retinoic acid metabolism. Annual review of nutrition 31: 65–87. 10.1146/annurev-nutr-072610-145127 PMC3789243 21529158

[16] Trautmann E, Guerquin MJ, Duquenne C, Lahaye JB, Habert R, et al (2008) Retinoic acid prevents germ cell mitotic arrest in mouse fetal testes. Cell cycle 7: 656–664. 18256537 10.4161/cc.7.5.5482

[17] Saga Y (2008) Sexual development of mouse germ cells: Nanos2 promotes the male germ cell fate by suppressing the female pathway. Development, growth & differentiation 50 Suppl 1S141–147. 10.1111/j.1440-169X.2008.01009.x 18430166

[18] Tsuda M, Sasaoka Y, Kiso M, Abe K, Haraguchi S, et al (2003) Conserved role of nanos proteins in germ cell development. Science 301: 1239–1241. 12947200 10.1126/science.1085222

[19] Suzuki A, Saga Y (2008) Nanos2 suppresses meiosis and promotes male germ cell differentiation. Genes & development 22: 430–435. 18281459 10.1101/gad.1612708 PMC2238665

[20] Li LB, Xia YK, Li XS, Lu J, Ma MF, et al (2008) [An analysis on chromosome X, Y and 18 in the spermatozoa of asthenospermia patients by triple-color fluorescence in situ hybridization]. Zhonghua nan ke xue = National journal of andrology 14: 211–214. 18488331

[21] Swain A (2006) Sex determination: time for meiosis? The gonad decides. Current biology : CB 16: R507–509. 16824913 10.1016/j.cub.2006.06.009

[22] Baltus AE, Menke DB, Hu YC, Goodheart ML, Carpenter AE, et al (2006) In germ cells of mouse embryonic ovaries, the decision to enter meiosis precedes premeiotic DNA replication. Nature genetics 38: 1430–1434. 17115059 10.1038/ng1919

[23] Revenkova E, Eijpe M, Heyting C, Hodges CA, Hunt PA, et al (2004) Cohesin SMC1 beta is required for meiotic chromosome dynamics, sister chromatid cohesion and DNA recombination. Nature cell biology 6: 555–562. 15146193 10.1038/ncb1135

[24] Mark M, Jacobs H, Oulad-Abdelghani M, Dennefeld C, Feret B, et al (2008) STRA8-deficient spermatocytes initiate, but fail to complete, meiosis and undergo premature chromosome condensation. Journal of cell science 121: 3233–3242. 18799790 10.1242/jcs.035071

[25] Ross AC (2003) Retinoid production and catabolism: role of diet in regulating retinol esterification and retinoic Acid oxidation. The Journal of nutrition 133: 291S–296S. 12514312 10.1093/jn/133.1.291S

[26] Wolf G (2001) Retinoic acid homeostasis: retinoic acid regulates liver retinol esterification as well as its own catabolic oxidation in liver. Nutrition reviews 59: 391–394. 11766909 10.1111/j.1753-4887.2001.tb06968.x

